# Double-hit of MIA and *Nod2* deficiency induces sex-specific offspring behavioral abnormalities through placental dysregulation

**DOI:** 10.1038/s41398-025-03747-z

**Published:** 2025-11-16

**Authors:** Zhen Cao, Xinyu Zhang, Fengjie Gao, Chuyao Wang, Zixuan Zhang, Jialu Jiang, Ningzhi Gou, Xiancang Ma, Yuan Gao

**Affiliations:** 1 Department of Psychiatry, The First Affiliated Hospital of Xi’an Jiaotong University, 277 Yanta West Road, Xi’an, 710061 China; 2 Center for Brain Science, The First Affiliated Hospital of Xi’an Jiaotong University, 277 Yanta West Road, Xi’an, 710061 China; 3 Shaanxi Provincial Key Laboratory of Biological Psychiatry, Xi’an, 710061 China

**Keywords:** Schizophrenia, Autism spectrum disorders

## Abstract

The pathogenesis of neuropsychiatric disorders, including autism spectrum disorder and schizophrenia, originates from complex interactions between genetic susceptibility and early environmental exposure. Infectious challenges during pregnancy are well-known environmental risk factors for neurodevelopmental disorders. Our previous research reported the interplay between maternal immune activation (MIA) and nucleotide-binding oligomerization domain-containing protein 2 (*Nod2*) signaling deficiency as potential genetic and environmental risk factors for schizophrenia pathogenesis. However, the mechanisms underlying this double-hit interaction—specifically regarding maternal-fetal interface homeostasis—remain unclear. In this study, we used the novel double-hit murine model that combines *Nod2* knockout (*Nod2*^−/−^) with polyinosinic:polycytidylic acid-induced MIA to systematically assess maternal metabolic profiles, placental developmental dynamics, and offspring behavioral phenotypes. We demonstrated that double-hit exposure has a significant effect on maternal metabolism and offspring development, characterized by sex-specific functional alterations in the placenta and increased susceptibility to neurodevelopmental disorders in male offspring. These results confirmed that the maternal environment modulates offspring neurodevelopment through placental mediation, highlighting the potential of modulating maternal immune-metabolic homeostasis as a preventive strategy against neurodevelopmental disorders.

## Introduction

The pathogenesis of neuropsychiatric disorders, including autism spectrum disorder (ASD) and schizophrenia, often originates from complex interactions between genetic susceptibility and early environmental exposure [1, 2]. Among environmental risk factors, gestational infection-triggered maternal immune activation (MIA) is a crucial determinant of offspring neurodevelopmental outcomes [3, 4]. Animal models have demonstrated that the administration of polyinosinic:polycytidylic acid (poly(I:C)) or lipopolysaccharides to pregnant dams can recapitulate the sterile immune response characteristics of MIA [5–7]. MIA disrupts maternal-fetal interface homeostasis, resulting in sustained immune alterations, behavioral abnormalities, and neurodevelopmental disorders in the offspring [8, 9].

Notably, not all maternal infections cause neurodevelopmental disorders in offspring. According to the multiple-hit model, the specificity of neurodevelopmental disorders arises from the synergistic effect of genetic susceptibility and environmental stressors during brain development [10]. Nucleotide-binding oligomerization domain-containing protein 2 (*Nod2*), a significant pattern recognition receptor (PRR) in innate immunity, participates in immune responses and is closely associated with metabolic homeostasis, neurodevelopment, and behavioral modulation [11, 12]. The comorbidity of schizophrenia and inflammatory bowel disease, as observed in epidemiological studies, is partially attributed to genetic overlap [13]. The loss-of-function mutation in *Nod2* is the strongest risk factor for Crohn’s disease. Therefore, we speculate that *Nod2* plays a crucial role in the gut-brain axis signal transduction. Although a previous study reported the synergistic effects of *Nod2* deficiency and MIA in eliciting schizophrenia-like behaviors and elevating maternal cytokines, including tumor necrosis factor-alpha and interleukin-17A, the underlying mechanisms require further assessment [14].

The maternal-fetal interface, comprising both the maternal decidua and embryonic placenta, constitutes a dynamic barrier that facilitates nutrient exchange, endocrine support, and immunomodulation [15, 16]. Accumulating evidence highlights the effect of placenta on fetal neurodevelopment by producing hormones and neurotransmitters, affecting immediate and long-lasting neurodevelopment through the placenta-brain axis [17, 18]. Placental dysfunction and barrier disruption are believed to contribute significantly to neurodevelopmental disorders, indicating a potential role in MIA-induced neurodevelopmental perturbations.

In this study, we primarily assessed the compounded effects of MIA and *Nod2* deficiency on embryonic neurodevelopment, with a specific emphasis on placental mediation. Our findings demonstrate that double-hit exposure induces profound metabolic dysregulation in pregnant dams and elicits sexually dimorphic behavioral abnormalities characterized by increased susceptibility in male offspring. Corresponding sex-specific functional alterations were identified in the placental tissue. These results confirm that the maternal environment modulates offspring neurodevelopment through placental mediation, highlighting the placenta as a primary target for understanding and potentially mitigating the risk of neurodevelopmental disorders. Our work will contribute to the development of novel treatments for preventing neurodevelopmental disorders by restoring homeostasis at the maternal-fetal interface.

## Materials and methods

### Mice

*Nod2* heterozygous (*Nod2*^+/−^) mice with a C57BL/6J background were obtained from Cyagen Biosciences, Inc. These mice were bred to produce *Nod2* knockout (*Nod2*^−/−^, KO) and wild-type (*Nod2*^+/+^, WT) littermates. Mice were housed in a temperature- and light-controlled specific pathogen-free facility (lights on/off at 07:00/19:00, 4–5 mice/cage), with ad libitum access to standard rodent chow and filtered water.

### MIA model establishment

Mice at 8–10 weeks of age were mated overnight, and females were checked every morning for seminal plugs and recorded as embryonic day 0.5 (E0.5). Pregnant mice were randomly administered intraperitoneal injections of either 20 mg/kg poly(I:C) to induce MIA or phosphate-buffered saline (PBS) as a negative control at embryonic day 12.5 (E12.5). In the first cohort, 12 *Nod2*^−/−^ dams were injected: two died during pregnancy; four received PBS (Total pups: 30; deaths: 3); and six received poly(I:C) (Total pups: 34; deaths: 4). Additionally, eight WT dams were injected: four received PBS (Total pups: 34; deaths: 0) and four received poly(I:C) (Total pups: 29; deaths: 2). These dams delivered naturally, and offspring were co-housed with their respective dams until weaning on postnatal day 21 (P21). Offspring were maintained for behavioral assessments on postnatal day 28 (P28) and between postnatal days 56–63 (P56–63), with both male and female progeny included in the test groups. The second cohort comprised six WT dams injected with PBS and six *Nod2*^−/−^ dams injected with poly(I:C). These dams were euthanized on embryonic day 14.5 (E14.5), and placental tissues and fetal brain specimens were collected for analysis.

### Behavioral tests

Based on sample size estimates from previous literature [19, 20], offspring that satisfied the pre-defined inclusion criteria (survival to P21, absence of physical abnormalities, and body weight within the normal range of control littermates) were included in the study. Consequently, a final cohort of n = 10 per group was established for behavioral analysis. The experimenters were blinded to the offspring identities during behavioral tests, and the test order was counterbalanced. Before testing, the mice were acclimatized to the test room for at least 1 h. Following each session, the arena was thoroughly cleaned with 75% ethanol to minimize olfactory cues.

### Marble-burying test (MBT)

The MBT was conducted in standard polypropylene mouse cages (40 × 25 × 18 cm) without lids. Fresh corncob bedding (5 cm depth) was leveled to create a uniform surface. Eighteen opaque glass marbles (15 mm diameter) were arranged in a symmetrical 6 × 3 grid. Each mouse was gently placed at the center of the cage. After each trial, marbles were considered buried when ≥ 50% of their surface was covered by bedding material. Marble index (%) = (buried marbles/total marbles) × 100.

### Open field test (OFT)

Each mouse was placed at the center of a standard testing arena (40 × 40 × 40 cm) for uniform positioning. Mouse behavior was recorded during a 30-minute session. The total distance traveled, time spent in the center zone (time in center %), and distance traveled within the center zone (distance in center %) were automatically analyzed using the Smart 3.0 video tracking system (Panlab, Spain).

### Three-chamber social test (TCST)

The TCST was conducted using a custom-built apparatus comprising three interconnected rectangular chambers (19 × 45 × 30 cm each) separated by Plexiglas dividers with central access gates (8 × 8 cm). Two identical wire-containment cages (7 × 7 × 14 cm) were positioned at the center of each side chamber. Before testing, the mice underwent a 10 min acclimation phase with free exploration of the entire apparatus containing empty cages. During the sociability test phase, mice were placed in the central chamber and allowed to explore both side chambers—one containing a wire cage with an age/sex-matched novel mouse and the other containing an empty wire cage (side randomly assigned). Social index (%) = (time spent sniffing the cage with mouse/total investigation time) × 100.

### Elevated plus maze test (EPM)

The EPM apparatus consisted of two open arms (30 × 5 cm) and two enclosed arms (30 × 5 × 15 cm) extending from a central platform (5 × 5 cm), elevated 50 cm above the floor. After a room acclimation, the mice were gently placed on the central platform facing an open arm, with video recording initiated after a 3 sec delay. The behavior was analyzed for 8 min using the Smart 3.0 video tracking system, with the software calculating the percentage of time spent exploring the open arms.

### Tissue collection and processing

On the morning of E14.5, pregnant mice were euthanized with 3–4% isoflurane. Maternal blood was collected via cardiac puncture, left at room temperature for 3 h, and then centrifuged (12,000 rpm, 10 min, 4 °C). The supernatant was aliquoted and stored at −80 °C. Placentas and fetuses were immediately collected and weighed, and the fetal brains were separated for further analysis. Placental efficiency was calculated as the fetal-to-placental weight ratio. A subset of placental and fetal brain samples was snap-frozen in liquid nitrogen and stored at −80 °C for subsequent quantitative polymerase chain reaction (qPCR) and RNA sequencing. The remaining placental samples were fixed overnight at 4 °C in 4% buffered paraformaldehyde for histological and immunohistochemical analyses.

### Gender identification

Genomic DNA was extracted from the caudal tissues of mouse embryos using the TSE014-Trelief® Mouse Direct PCR Kit (TSINGKE, China). Embryonic sex determination was performed by PCR amplification of the sex-determining region Y chromosome (*Sry*) gene using specific primers: 5’-ACAAGTTGGCCCAGCAGAAT-3’ (forward) and 5’-GGGATATCAACAGGCTGCCA-3’ (reverse), following the manufacturer’s protocol. Amplification products were electrophoresed on 1% agarose gels and visualized under UV transillumination. Genetic sex was determined based on the presence or absence of the *Sry* band.

### Histology and hematoxylin and eosin (H&E) staining

Paraformaldehyde-fixed placental tissues were dehydrated using a graded ethanol series and embedded in paraffin. Serial 5 μm sections were dewaxed in xylene, rehydrated through a descending ethanol series, and stained with H&E for nuclear and cytoplasmic visualization, respectively. Subsequently, the sections were dehydrated in graded ethanol, permeabilized with xylene, and mounted using neutral gum. High-resolution images were captured using a scanner (Dx12; 3DHISTECH Ltd., Hungary).

For morphometric analysis, six midsagittal fields from each placenta were analyzed. The junctional zone and labyrinth layers of the placenta were quantified using Fiji/ImageJ (NIH, USA), and their maximum areas were calculated from two serial sections per individual [21].

### Immunofluorescence staining

Paraffin-embedded tissue sections were dewaxed in xylene, rehydrated through a graded ethanol series, and subjected to antigen retrieval by heating in sodium citrate buffer (BL619A; Biosharp) at 95 °C for 15 min. After cooling, the sections were washed thrice with PBS and permeabilized with 0.5% Triton X-100 (T8200; Solarbio) for 1 h at room temperature. After three PBS washes, blocking was performed with normal goat serum (AR0009; Boster) for 1 h at room temperature, followed by three PBS washes.

Subsequently, the sections were incubated separately with primary antibodies against cytokeratin 7 (*CK7*) (1:400, 17513-1-AP; Proteintech) and cluster of differentiation 31 (*CD31*) (1:400, 28083-1-AP; Proteintech), each diluted in PBS, overnight at 4 °C. After washing thrice with PBS, the sections were incubated with the corresponding fluorescent secondary antibodies—Coralite488-conjugated goat anti-rabbit IgG (H + L) (1:100, SA00013-2; Proteintech) and Coralite594-conjugated goat anti-rabbit IgG (H + L) (1:100, SA00013-4; Proteintech)—for 2 h at room temperature in the dark. The sections were washed thrice with PBS, counterstained with 4′,6-diamidino-2-phenylindole (DAPI) (AR1176; Boster) for 10 min at room temperature, and rinsed thrice with PBS. Finally, the slides were mounted with an antifade medium (AR1109; Boster). The images were acquired using an Olympus FV3000 microscope (Olympus, Japan), and fluorescence intensity was analyzed using Fiji/ImageJ (NIH, USA). High-resolution images were acquired using a Pannoramic MIDI scanner (3DHISTECH Ltd., Hungary).

### Real-time qPCR

Complementary DNA was synthesized from total RNA extracted from the placentas and fetal brains using the M5 Universal RNA mini kit (Mei5bio) and reverse-transcribed using the PrimeScript™ FAST RT reagent Kit with gDNA Eraser (TaKaRa). RNA quality and yield were assessed using a DA-11 Spectrophotometer (DeNovix, USA). Real-time qPCR was performed on a CEX Connect™ Real-Time System (Bio-Rad, USA) using BeyoFast™ SYBR Green qPCR Mix (D7262; Beyotime). PCR was performed using primer pairs targeting hypoxanthine phosphoribosyltransferase 1 (*Hprt*), apolipoprotein E (*Apoe*), insulin-like growth factor 2 (*Igf2*), lipocalin 2 (*Lcn2*), leptin receptor (*Lepr*), vascular endothelial growth factor A (*Vegfa*), C-C motif chemokine ligand 2 (*Ccl2*), C-C motif chemokine receptor 2 (*Ccr2*), interferon-induced protein with tetratricopeptide repeats 1 (*Ifit1*), *Ifit3*, MX dynamin-Like GTPase 1 (*Mx1*), Toll-like receptor 7 (*Tlr7*), myocyte enhancer factor 2 C (*Mef2c*), neuronal differentiation 2 (*Neurod2*), nuclear factor I B (*Nfib*), plexin A4 (*Plxna4*), solute carrier family 17 member 7 (*Slc17a7*), and zinc finger E-box binding homeobox 2 (*Zeb2*) (Supplementary Table 1). The reaction volume was 20 µL per well. Experiments were performed in triplicate, and the results were normalized to *Hprt* expression. The expression level of each sample was presented as 2^−ΔΔCt^.

### RNA sequencing (RNA-seq) and analysis

Total RNA was extracted from frozen placental tissue using the TRIzol method, following the manufacturer’s protocol (Novogene, China). RNA integrity was assessed using the RNA Nano 6000 Assay Kit of the Bioanalyzer 2100 system (Agilent Technologies, CA, USA). High-quality RNA was purified and subjected to PCR amplification for library preparation. The PCR products were purified using the AMPure XP system, and the final library quality was assessed using the Agilent Bioanalyzer 2100 system. Clustering was performed on a cBot system using the TruSeq PE Cluster Kit v3 (Illumina), followed by 150 bp paired-end sequencing on the Illumina NovaSeq platform.

Raw RNA-seq data in fastq format were quality-filtered using fastp (v0.23.2) to remove adapters, poly N sequences, and low quality reads. Clean data were assessed for Q20, Q30, and GC content before the downstream analysis. Differential expression analysis was performed between groups using DESeq2 (v1.20.0). Differentially expressed genes (DEGs) were defined as those with a fold change (FC) of ≥ 1.5 or ≤ 0.67 (corresponding to |log_2_FC| ≥ 0.585) and a *P* < 0.05. Gene Ontology (GO) and Kyoto Encyclopedia of Genes and Genomes (KEGG) enrichment analyses of DEGs were performed using clusterProfiler (v4.0.5), with pathways considered significantly enriched at adjusted *P* < 0.05 (Benjamini–Hochberg method).

### Untargeted metabolomics and analysis

Maternal serum was thawed and extracted using an acetonitrile: methanol solution containing internal standards. After centrifugation, the supernatants were collected and prepared for liquid chromatography–mass spectrometry analysis. Chromatographic separation was performed on a Waters ACQUITY Premier HSS T3 column (1.8 μm, 2.1 × 100 mm) using 0.1% formic acid in water (mobile phase A) and acetonitrile (mobile phase B). A gradient elution program was used, and analyses were conducted in both the positive and negative electrospray ionization (ESI) modes. The column was maintained at 40 °C, with a flow rate of 0.4 mL/min and an injection volume of 4 μL. Mass spectrometry was performed using ESI in both ionization modes, alternating between full-scan and data-dependent MSn acquisition (with dynamic exclusion) over an m/z range of 75–1000 at a resolution of 35,000.

Differential metabolites (DEMs) were identified by combining orthogonal partial least squares discriminant analysis (OPLS-DA) and univariate statistical testing. Metabolites with variable importance in projection (VIP) ≥ 1.5 (from a permutation-validated OPLS-DA model) and *P* < 0.05 were considered statistically significant DEMs. Before modeling, the data were log_2_-transformed and mean-centered. To prevent overfitting, model robustness was assessed using a 200-iteration permutation test. Subsequently, the identified metabolites were structurally annotated against the KEGG compound database and functionally contextualized via pathway mapping using the KEGG pathway database.

### Statistical analysis

Statistical analyses were performed using R software (version 4.4.3; R Foundation for Statistical Computing, Vienna, Austria). Data are presented as the mean ± standard error of the mean (SEM). The Shapiro–Wilk test was used to assess the normality of the data distribution, and Levene’s test was used to assess variance homogeneity. For two-group comparisons, the unpaired Student’s *t*-test was used when both normality and homogeneity assumptions were met; otherwise, the non-parametric Wilcoxon rank-sum test was applied. For multiple group comparisons, one-way analysis of variance followed by Tukey’s honest significant difference post hoc test was used for normally distributed data with equal variances. When the data failed to meet these assumptions, the Kruskal–Wallis test was performed, followed by Dunn’s post hoc test for pairwise comparisons. Statistical significance was set at *P* < 0.05. Statistical significance is indicated in the figures as follows: *P < 0.05, **P < 0.01, and ***P < 0.001.

## Results

### Double-hit of *Nod2* KO and MIA induces behavioral abnormalities in offspring

**Fig. 1:**
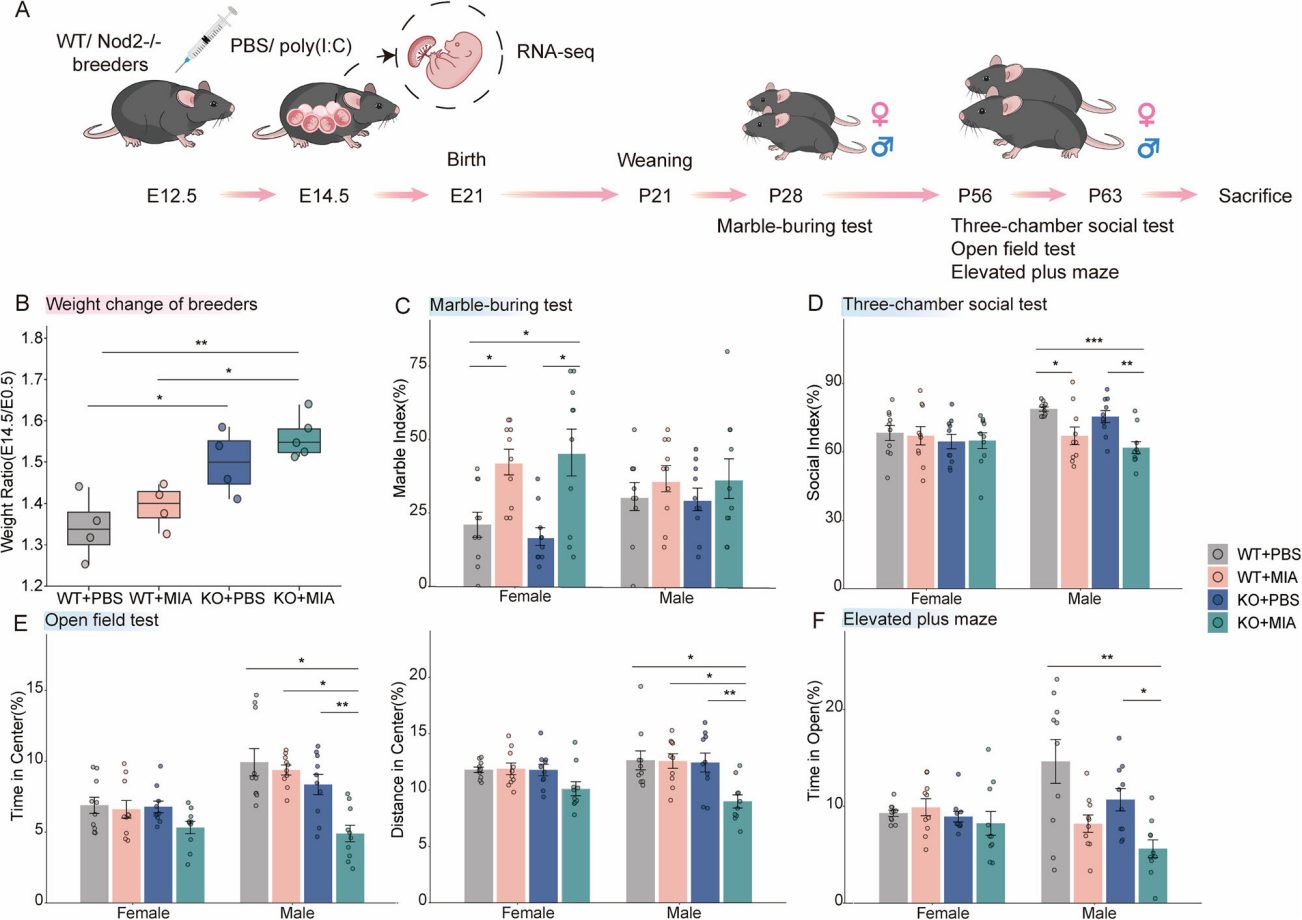
Experimental overview and behavioral outcomes. **A** Schematic illustration of maternal immune activation (MIA) paradigm. Wild-type (WT) and nucleotide-binding oligomerization domain-containing protein 2 knockout (*Nod2*^−/−^) breeders received intraperitoneal polyinosinic:polycytidylic acid (poly(I:C)) (20 mg/kg) or phosphate-buffered saline (PBS) at embryonic day (E12.5). Fetal tissues (brain/placenta) collected at E14.5 for RNA sequencing. Offspring behavioral tests: Marble-burying test (MBT) at postnatal day (P28), social/exploratory assays between P56 and P63. **B** Box plot of maternal weight at E14.5 to E0.5 (n = 4–5 per group). Data are presented as the median (center line), the upper and lower quartiles (bounds of box), and the range (whiskers). **C** MBT. Marble index (%) = (the number of buried marbles in 30 min/total number) × 100. **D** Three-chamber social test. Social index (%) = (time spent sniffing the cage with mouse/total investigation time) × 100. **E** Open field test. Time spent in and distance traveled in the center of the arena (%). **F** Elevated plus maze test. Open arm exploration time (%). The offspring sample size: n = 10 per group. Group definitions: WT + PBS: wild type dams + PBS treatment; WT + MIA: wild type dams + poly(I:C) treatment; KO + PBS: *Nod2*^−/−^ dams + PBS treatment; and KO + MIA: *Nod2*^−/−^ dams + poly(I:C) treatment. Behavioral data expressed as mean ± SEM; one-way ANOVA followed by Tukey’s post-hoc and Kruskal–Wallis tests followed by Dunn’s multiple comparisons test (*P < 0.05, **P < 0.01, and ***P < 0.001).

The experimental design is illustrated in Fig. 1A. Maternal body weight exhibited no significant differences among groups at E12.5 (Fig. S1A). However, 48 h post-intervention, dams in the double-hit KO + MIA group exhibited significantly higher weight than that in the WT + PBS group (Fig. 1B). Behavioral phenotyping revealed that MIA significantly increased stereotypic behavior in female offspring, independent of genotype, in the marble-burying test, whereas no such effect was observed in males (Fig. 1C). During social and anxiety tests (TCST, OFT, and EPM), male offspring from the WT + MIA group exhibited social deficits, whereas the double-hit males exhibited exacerbated social deficits accompanied by significantly heightened anxiety-like behavior (Fig. 1D–F). These findings demonstrate sexual dimorphism in MIA-induced neurodevelopmental effects and indicate that *Nod2* deficiency synergistically exacerbates MIA’s adverse outcomes, with this synergy being specifically significant in male offspring.

### Metabolic disturbances in double-hit dams

**Fig. 2:**
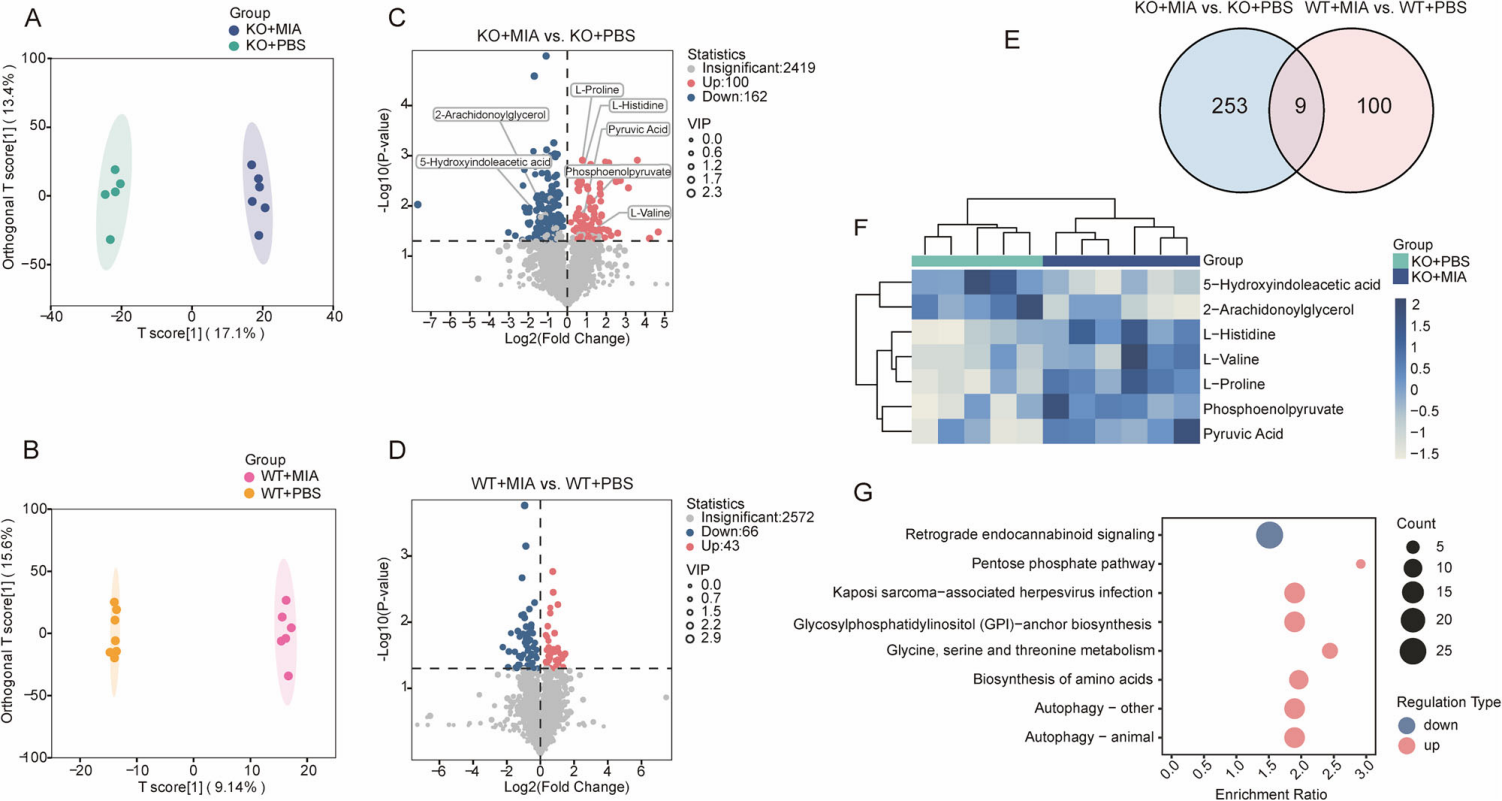
Serum untargeted metabolomics reveals maternal metabolic disturbances. **A** Orthogonal partial least squares discriminant analysis (OPLS-DA) of KO + MIA vs. KO + PBS. **B** OPLS-DA analysis of WT + MIA vs. WT + PBS. **C** Volcano plot for KO + MIA vs. KO + PBS (*P* < 0.05 and VIP ≥ 1.5). **D** Volcano plot for WT + MIA vs. WT + PBS (same threshold). **E** Venn diagram of shared/unique differential metabolites (DEMs) between comparisons. **F** Cluster heatmap of primary metabolites in KO + MIA vs. KO + PBS. **G** Kyoto Encyclopedia of Genes and Genomes pathway enrichment of *Nod2*^−/−^ specific DEMs. Sample size: n = 6 per group.

Aberrant maternal weight gain indicated disrupted metabolic homeostasis (Fig. 1B) that may increase the risk of cognitive impairment in offspring [22]. To assess the potential effects of *Nod2*^−/−^ and MIA on maternal metabolites, we collected serum samples after poly(I:C) injection and conducted untargeted metabolomic analysis. OPLS-DA revealed an obvious metabolic separation between the KO + MIA and KO + PBS groups (permutation test: Q² = 0.593, *P* < 0.05; Fig. S2A), whereas the WT groups exhibited significant metabolic overlap (Q^2^ = −0.0418, *P* = 0.915; Fig. S2B), indicating that MIA significantly disrupts the serum metabolome—specifically under *Nod2* deficiency (Fig. 2A). DEM screening (*P* < 0.05, VIP ≥ 1.5) identified 262 DEMs (100 upregulated and 162 downregulated) induced by MIA in *Nod2*^−/−^ dams—exceeding the 109 DEMs (43 upregulated and 66 downregulated) in WT dams (Fig. 2C, D). Venn analysis confirmed only nine shared DEMs across genotypes (Fig. 2E), confirming that *Nod2* deficiency uniquely reprogrammed the MIA-responsive metabolic network. Hierarchical clustering heatmap of primary metabolites (Fig. 2F) and KEGG enrichment (Fig. 2G) of *Nod2*^−/−^ specific DEMs highlighted significant pathway enrichment in biosynthesis of amino acids, retrograde endocannabinoid signaling, glycoylphosphatidylinositol-anchor biosynthesis, autophagy, and pentose phosphate pathway.

### Double-hit placentas exhibit structural disruption

**Fig. 3:**
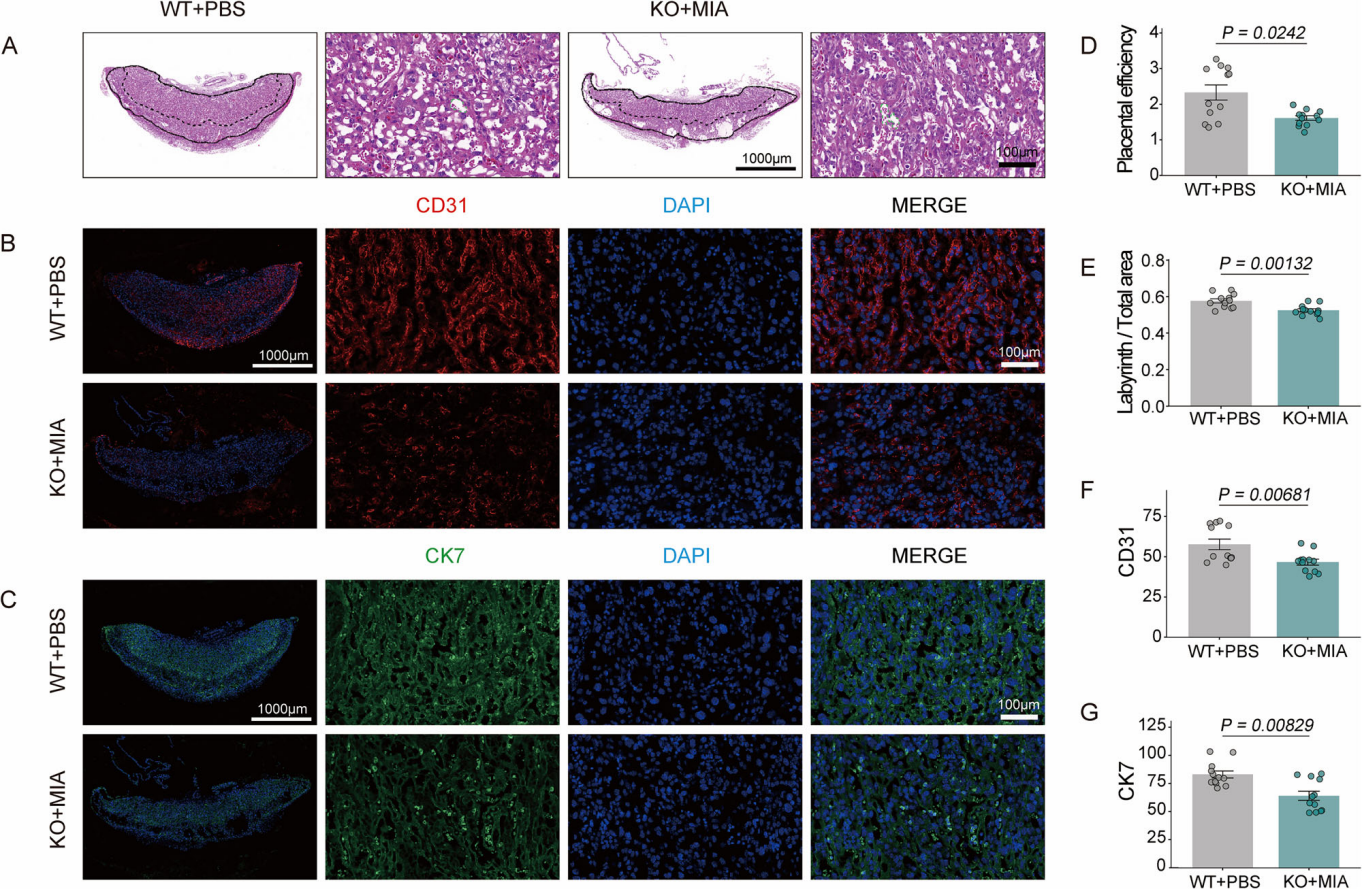
Morphological and functional analysis of placental development. **A** Hematoxylin and eosin staining of placental tissue. Left: Overall structure (Scale bar: 1000 μm); Right: Magnification of sinusoids (Scale bar: 100 μm). **B** Immunofluorescence for cluster of differentiation 31 (*CD31*) (red) and 4′,6-diamidino-2-phenylindole (DAPI) (blue). From left to right: Overview (Scale bar: 1000 μm), *CD31* channel, DAPI channel, and merged image (Scale bar: 100 μm). **C** Immunofluorescence for cytokeratin 7 (*CK7*) (red) and DAPI (blue). Panels arranged as in B. **D** Comparison of placental efficiency (fetal-to-placental weight ratio) between groups. **E** Ratio of labyrinth zone to total area (labyrinth + junction zone). **F** Quantification of *CD31* fluorescence intensity. **G** Quantification of *CK7* fluorescence intensity. Sample size: n = 12 per group (male: female = 1:1). Data expressed as mean ± SEM; Student’s *t*-test or the non-parametric Wilcoxon rank-sum test.

Because the placenta serves as the primary site of maternal-fetal interaction, its functional status is intrinsically associated with fetal growth dynamics. To determine whether the observed behavioral abnormalities were associated with a loss of placental efficiency, we assessed the fetal-to-placental weight ratio, defined as grams of fetus produced per gram of placenta. Notably, placental efficiency was significantly reduced in the double-hit group at E14.5 (Fig. 3D), indicating a potential functional compromise. H&E staining further revealed significant structural alterations, characterized by a reduced labyrinth/total area ratio and fewer sinusoids (Fig. 3A and E). Additionally, double-hit exposure downregulated primary functional markers—angiogenesis indicator *CD31* and spiral artery remodeling marker *CK7* [23] (Fig. 3B, C, F and G). These findings indicate that double-hit exposure disrupts placental function by affecting endothelial development and angiogenesis, highlighting placental mechanisms as contributors to the maternal origins of offspring neurodevelopmental disorders.

### Overall and Sex-Specific transcriptional alterations in placentas

**Fig. 4:**
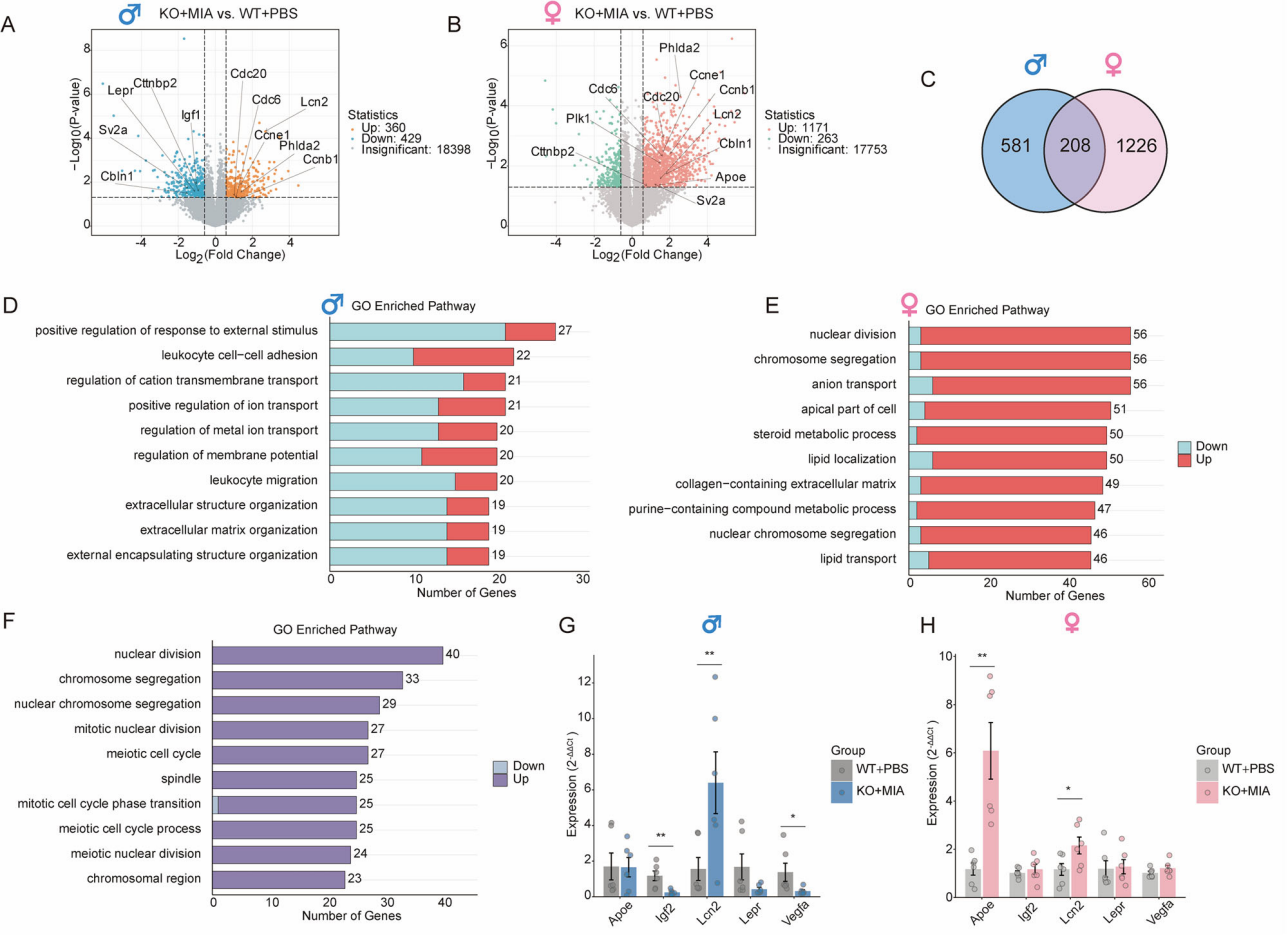
Both shared and sex-specific alterations exist in male and female placentas. **A** Male placental differentially expressed genes (DEGs) (KO + MIA vs. WT + PBS; |log_2_FC| ≥ 0.585 and *P* < 0.05; n = 5–6 per group). **B** Female placental DEGs (KO + MIA vs. WT + PBS; same threshold). **C** Venn diagram of shared/unique DEGs between comparisons. **D** Top 10 enriched Gene Ontology (GO) terms of male-specific DEGs ranked by gene count. **E** Top 10 enriched GO terms of female-specific DEGs ranked by gene count. **F** Top 10 enriched GO terms of shared DEGs ranked by gene count. **G** Quantitative polymerase chain reaction (qPCR) validation of primary male DEGs. Expression levels are normalized to *Hprt* and presented as 2^−ΔΔCt^ values (n = 6). **H** qPCR validation of female primary DEGs (same analysis). Data expressed as mean ± SEM; Student’s *t*-test or the non-parametric Wilcoxon rank-sum test (*P < 0.05, **P < 0.01, and ***P < 0.001).

Because of the structural disruption of placenta by double-hit exposure and its behavioral sexual dimorphism, we performed sex-stratified transcriptomic analysis of the placentas in *Nod2*^−/−^ mice receiving poly(I:C) vs. PBS-exposed WT mice. The results demonstrated 789 DEGs in double-hit male placentas (360 upregulated and 429 downregulated), whereas double-hit females exhibited 1434 DEGs (1171 upregulated and 263 downregulated) (Fig. 4A, B). Venn analysis identified 208 shared DEGs, of which 201 demonstrated concordant expression trends (Fig. 4C). Notably, neurodevelopment-associated genes (cortactin-binding protein 2 [*Cttnbp2*], synaptic vesicle glycoprotein 2 A [*Sv2a*], and cerebellin 1 precursor protein [*Cbln1*]) were downregulated in males but upregulated in females. GO enrichment analysis of the shared 201 DEGs revealed significant upregulation of cell cycle regulation pathways (nuclear division and chromosome segregation) (Fig. 4F).

### Double hit induces sex-specific transcriptional deregulation in placental and fetal brain

Further analyses revealed sex-specific gene expression characterized by double-hit exposure. Male-specific 581 DEGs were enriched in the positive regulation of response to external stimuli and ion transport. These DEGs were primarily downregulated, indicating immune dysregulation and transport deficits (Fig. 4D). Female-specific 1226 DEGs were enriched in anion transport and lipid transport—that were overall upregulated, indicating enhanced metabolic adaptation to stress (Fig. 4E). qPCR validation confirmed the significant upregulation of *Lcn2* in both sexes and female-specific *Apoe* upregulation, consistent with the RNA-seq analysis. (Fig. 4G, H). Notably, qPCR revealed the downregulation of *Igf2* and *Vegfa* in the male placentas (not detected by RNA-seq), highlighting the increased vulnerability of male placenta to double-hit exposure.

**Fig. 5:**
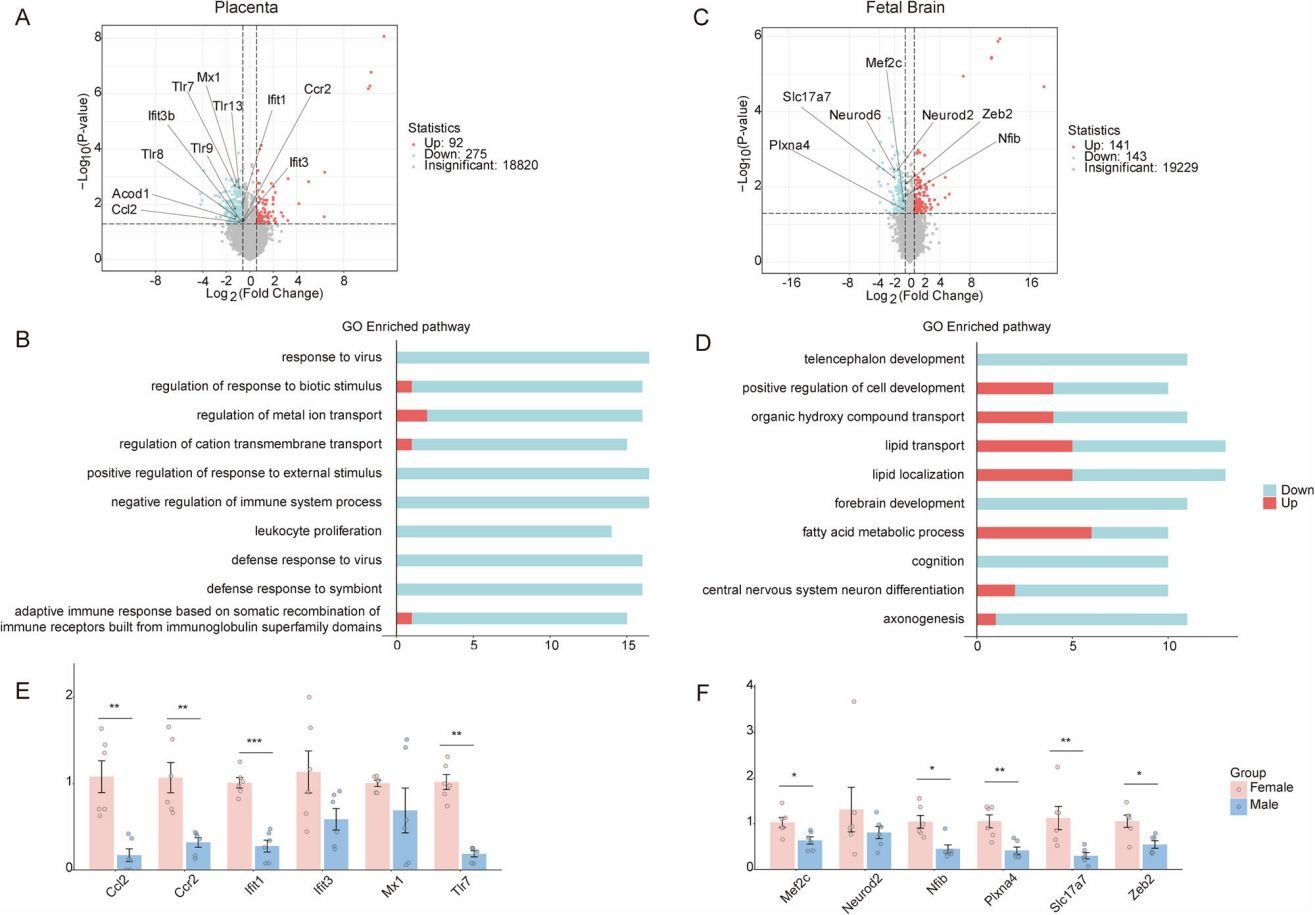
Transcriptomic sex differences in double-hit placentas and fetal brains. **A** Volcano plot of DEGs in KO + MIA male vs. female placentas (|log_2_FC| ≥ 0.585 and *P* < 0.05). **B** Top 10 enriched GO terms of DEGs ranked by gene count. **E** qPCR validation of primary DEGs. Expression normalized to *Hprt* and presented as 2^−ΔΔCt^ values (n = 6). **C,** **D, F** Corresponding analysis to **A**, **B**, **E** for fetal brains. Data expressed as mean ± SEM; Student’s *t*-test or the non-parametric Wilcoxon rank-sum test (*P < 0.05, **P < 0.01, and ***P < 0.001).

To further elucidate placental sexual dimorphism, we directly compared the transcriptomes of double-hit male vs. female placentas, identifying 367 sex-dependent DEGs (92 upregulated and 275 downregulated in males) (Fig. 5A). Notably, the downregulated DEGs included primary interferon pathway regulators (*Tlr7*, *Tlr9*, *Ifit1*, and *Mx1*), and GO analysis revealed broad downregulation of viral response and immune regulation pathways (Fig. 5B).

To assess the mechanisms by which maternal metabolic disorders contribute to adverse behavioral outcomes in the offspring, we performed transcriptomic profiling of the fetal brains of *Nod2*^−/−^ mice 48 h after poly(I:C) injection. Because of sexual dimorphism in offspring behavior, parallel fetal brain transcriptomic analysis identified 284 sex-dependent DEGs (141 upregulated and 143 downregulated in males), among which the downregulated genes included primary regulatory genes for neurodevelopment (*Neurod2*, *Neurod6*, and *Nfib*) (Fig. 5C). Pathway enrichment analysis demonstrated significant downregulation of telencephalon development and cognition in the male fetal brains (Fig. 5D), consistent with their more severe behavioral impairments. qPCR validation confirmed concordant expression trends for placental immune and fetal brain developmental genes with sequencing data (Fig. 5E and F).

## Discussion

This study reports a double-hit model combining poly(I:C)-induced MIA with *Nod2* KO, revealing sex-specific mechanisms by which maternal immune metabolic dysregulation affects fetal neurodevelopment through the placenta. The double-hit induced anxiety-like behavior and social deficits in male offspring, whereas females exhibited increased early stereotyped behaviors. In mothers, double-hit dams demonstrated abnormal weight gain and altered serum metabolomic profiles. These alterations were reflected at the maternal-fetal interface and may affect offspring development [24], increasing the risk of metabolic and neuropsychiatric disorders. Placental pathology revealed reduced efficiency, labyrinth atrophy, and impaired angiogenesis, indicating generalized dysfunction. Transcriptomic profiling demonstrated shared disruptions in cell cycle regulation, with sex-specific alterations in transport function—downregulated in males and upregulated in females. In male fetuses, neurodevelopmental and cognitive-related pathways were broadly downregulated. This was accompanied by the defects of innate immune pathways in the male placenta, providing a cross-organ mechanism underlying increased male vulnerability to genetic and environmental interactions.

Neuropsychiatric disorders, such as ASD and schizophrenia are more prevalent in males [25, 26], and previous MIA studies have similarly reported increased male susceptibility [7, 27]. Consistently, as demonstrated in our previous work where double-hit male offspring exhibited more severe schizophrenia-like behaviors, including prepulse inhibition deficits [14], the current study also revealed more pronounced behavioral alterations in males. In contrast, females showed relative resilience. We hypothesize that this sex-specific divergence is potentially mediated by the placenta. Transcriptome analysis revealed significant downregulation of primary antiviral defense pathways in male placentas, particularly involving toll-like receptor family members (*Tlr7*, *Tlr8*, *Tlr9*, and *Tlr13*) and interferon-stimulated genes (ISGs, *Mx1*, *Ifit1*, and *Ifit3*) [28]. Because PRR- and ISG-mediated type I interferon signaling is essential for innate antiviral immunity [29], its impairment in dams and fetuses typically increases the rates of maternal, placental and fetal infection [30]. Additionally, male-specific downregulation of aconitate decarboxylase 1 (*Acod1*) and *Ccl2/Ccr2*, genes associated with impaired anti-infection protection [31] and placental leukopenia following maternal stress [32], respectively, was observed, indicating immune dysregulation and compromised anti-infection capacity.

This placental vulnerability was mirrored in male fetal brains, demonstrating the downregulation of primary neurodevelopmental genes, such as *Mef2c*, *Neurod2*, *Neurod6*, and *Zeb2. Mef2c* is thought to be associated with cognitive deficits and increased schizophrenia risk in patients [33]. *Neurod2* and *Neurod6* are crucial for normal central nervous system development, function, and the survival of specific neuronal subtypes [34]. Additionally, *Zeb2* regulates diverse neurodevelopmental processes, and its dysfunction is associated with neurodevelopmental disorders [35]. The downregulation of relevant GO (telencephalon development and cognition) and KEGG pathways (Neuroactive ligand-receptor interaction; Fig. S5B) further confirmed male neurodevelopmental dysregulation. Although no direct evidence has confirmed a causal relationship between the downregulation of placental immune responses and fetal brain development, our results support the hypothesis that placental immune defects may mediate offspring neurodevelopmental abnormalities through the placenta-fetal brain axis.

Placental size, structure, and fetal interactions are crucial for nutrient exchange and optimal fetal growth. A low fetal-to-placental weight ratio indicates inefficient nutrient transport and is associated with increased pregnancy complications [36]. We demonstrated that maternal metabolic disorders, induced by double-hit model, significantly affect fetal resorption at E14.5. This was confirmed by a reduction in the labyrinth zone—the placenta’s primary site for maternal-fetal exchange. Impaired efficiency, structural pathology, and molecular dysregulation in the placentas collectively indicate a global functional compromise induced by the combined effects of *Nod2* KO and MIA. Both sexes exhibited upregulation of the immune-pro-inflammatory gene (*Lcn2*) and the growth-restricting imprinted gene Pleckstrin homology-like domain, family A, member 2 (*Phlda2*), indicating shared placental damage and its direct effect on offspring development [37, 38].

Additionally, our data indicated that placental responses exhibit significant sexual dimorphism in the double-hit model. Shared genes downregulated in males and upregulated in females included primary neurodevelopment-associated genes, such as the synaptic organizer (*Cbln*) (whose impairment causes social deficits) [39], synaptic scaffolding protein *Cttnbp2* (whose deficiency causes autism-like behavior) [40], and synaptic vesicle protein *Sv2a* (with its reduction observed in schizophrenia) [41]. At the pathway level, male placentas exhibited suppressed ion transport, whereas female placentas exhibited enhanced proliferation and upregulation of ion and lipid transport. Because ion channels are essential for hormone synthesis, trophoblast homeostasis, micronutrient transfer, and vascular regulation [42], their dysfunction directly affects placental efficiency and fetal development. Female placentas may buffer environmental stress through lipid accumulation [43]. In this study, the upregulation of ion/lipid transport in female placentas may represent a protective adaptation that alleviates neurodevelopmental impairment. Additionally, double-hit male-specific downregulation of *Vegfa* [44] and *Igf2* [45] further indicates impaired angiogenesis and nutrient delivery, supporting the hypothesis that sex-specific placental MIA responses contribute to differential offspring vulnerability [46, 47].

MIA amplifies genetic risk through metabolic-immune interactions. The double-hit challenge triggered significant maternal serum metabolic disruption, characterized by dysregulated amino acid biosynthesis/metabolism, downregulated retrograde endocannabinoid (eCB) signaling, and increased energy metabolism. These metabolic alterations directly impair placental and fetal development. In particular, aberrant histidine metabolism may impair placental perfusion by modulating diamine oxidase activity [48], whereas increased proline levels are associated with excitotoxicity and neuroinflammation [49]. 2-Arachidonoylglycerol (2-AG), a primary component of the eCB system, plays an essential role in neurodevelopment during early gestation, affecting the uterus, placenta, and fetal brain [50]. Imbalances in 2-AG levels are associated with schizophrenia-like behaviors [51]. Additionally, as a member of the arachidonic acid cascade, reduced 2-AG levels are associated with impaired anti-inflammatory responses [52]. Concurrently, reduced 5-hydroxyindoleacetic acid levels indicate tryptophan metabolic dysregulation and are associated with diabetic metabolic disorders [53]. Therefore, our findings confirm that maternal environmental stressors affect fetal development through placental adaptations, consistent with previous studies [54].

Despite the novel findings presented above, acknowledging the limitations of this study is essential. First, the causal relationship between placental dysfunction and offspring brain development would benefit from further validation using interventional strategies. Second, the interaction between maternal metabolomic profiles and placental transcriptomics has not been comprehensively explored, and the broader regulatory network encompassing the maternal–placental–fetal brain axis remains to be fully elucidated. Third, although this study focused on placental mechanisms, the double-hit model likely affects neurodevelopment through multiple additional pathways, including gut microbiota alterations previously documented in this model [14].

In summary, we integrated multi-level data across maternal metabolism, placental and fetal development, and offspring behavior, systematically illustrating how the double challenge of *Nod2*^−/−^ and MIA disrupts neurodevelopment in a sex-specific manner. This highlights the crucial regulatory role of sex-dimorphic placental responses in determining offspring vulnerability. Future studies should include causal intervention experiments to verify the placental-fetal brain association and explore the dynamic relationship between maternal metabolism and placental gene expression. Multi-omics integration and functional validation are essential to identify crucial developmental events and ultimately translate these findings into clinically viable sex-specific preventive strategies.

## Supplementary information

Supplement information

## Data Availability

The raw sequence data reported in this paper have been deposited in the Genome Sequence Archive (Genomics, Proteomics & Bioinformatics 2021) in National Genomics Data Center (Nucleic Acids Res 2022), China National Center for Bioinformation / Beijing Institute of Genomics, Chinese Academy of Sciences (GSA: CRA027848) that are publicly accessible at https://ngdc.cncb.ac.cn/gsa.
