# Supplementary material for: Double-hit of MIA and Nod2 deficiency induces sex-specific offspring behavioral abnormalities through placental dysregulation
Source: Transl Psychiatry. 2025 Nov 16;15:527. doi: 10.1038/s41398-025-03747-z (PMC12712060; doi:10.1038/s41398-025-03747-z)
Supplement: Supplementary file 1 — Supplement information [file 41398_2025_3747_MOESM1_ESM.docx]

Table S1: Primer sequences

| **Gene** | **Forward primer** | **Reverse primer** |
| --- | --- | --- |
| *Hprt* | CTGGTGAAAAGGACCTCTCGAAG | CCAGTTTCACTAATGACACAAACG |
| *Apoe* | CAGAGCTCCCAAGTCACACA | AGTCGGTTGCGTAGATCCTC |
| *Igf2* | GTCGCATGCTTGCCAAAGAG | GGTGGTAACACGATCAGGGG |
| *Lcn2* | AGGACGACAACATCATCTTCTC | TGGAGTGGCAGACAGACAG |
| *Lepr* | GCTCTTCTGATGTATTTGGAAATC | ACCTGATATTGAAGCGGAAATGG |
| *Vegfa* | CCACGTCAGAGAGCAACATCA | TCATTCTCTCTATGTGCTGGCTTT |
| *Ccl2* | CAGGTCCCTGTCATGCTTCT | GTCAGCACAGACCTCTCTCT |
| *Ccr2* | GCTCTACATTCACTCCTTCCAC | ACCACTGTCTTTGAGGCTTG |
| *Ifit1* | CTCTGAAAGTGGAGCCAGAAAAC | AAATCTTGGCGATAGGCTACGA |
| *Ifit3* | TGAACTGCTCAGCCCACA | TCCCGGTTGACCTCACTC |
| *Mx1* | AAACCTGATCCGACTTCACTTCC | TGATCGTCTTCAAGGTTTCCTTGT |
| *Tlr7* | ATGTGGACACGGAAGAGACAA | GGTAAGGGTAAGATTGGTGGTG |
| *Mef2c* | CCATTGGACTCACCAGACCT | AGCACACACACACACTGCAA |
| *Neurod2* | AGAAGCTGTCCAAGATCGAGAC | CCGTGAGGAAGTTACGAGAGTT |
| *Nfib* | AGAAGCCCGAAATCAAGCAGA | GCCAGTCACGGTAAGCACAAA |
| *Plxna4* | ACAGGGCACATTTATTTGGGG | CACTTGGGGTTGTCCTCATCT |
| *Slc17a7* | GTGCAATGACCAAGCACAAG | AGATGACACCGCCGTAGTG |
| *Zeb2* | GGAAGAGTTGATGCCACGGT | TCCGTCAAGTCACAGGGCTC |


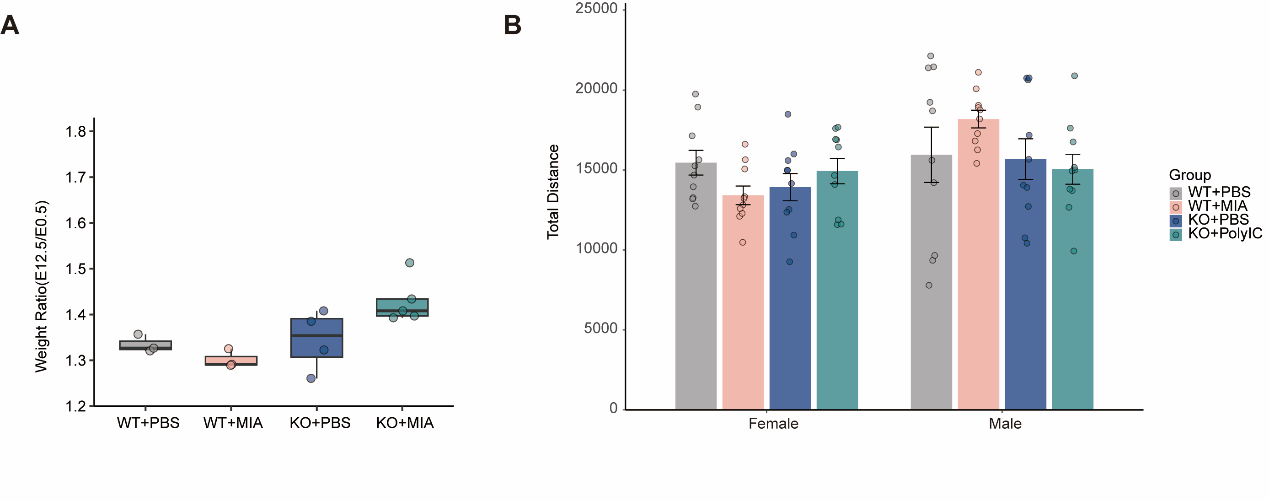


Fig. S1. Maternal weight and offspring behavior. (A) Box plot of maternal weight at E12.5 to E0.5 (n = 4–5). Data are presented as the median (center line), the upper and lower quartiles (bounds of box), and the range (whiskers). (B) Open field test. Total distance traveled by mice in the open field arena during 30 min (n = 10). Data expressed as mean ± SEM; one-way ANOVA, differences not significant (*P* > 0.05).


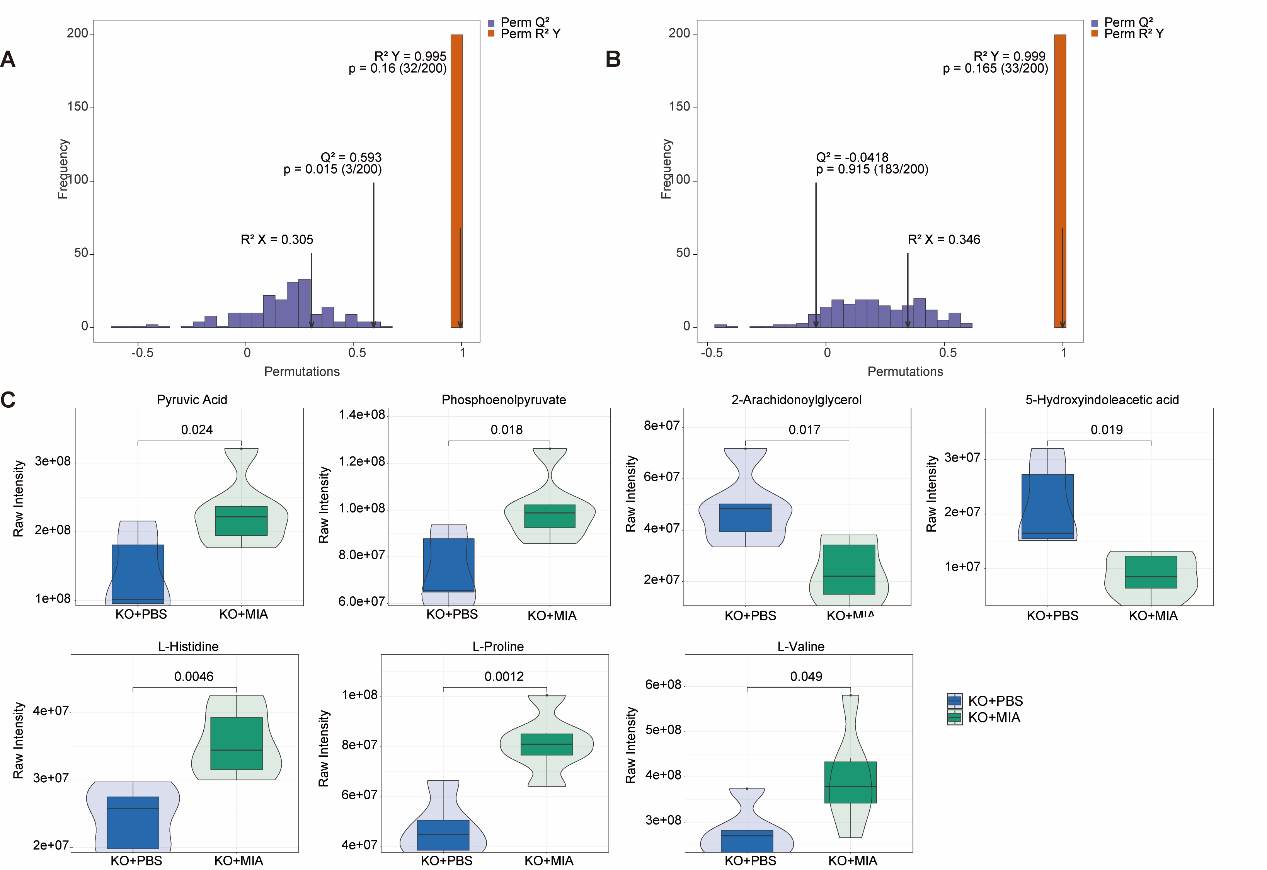


Fig. S2. Validations of OPLS-DA models and primary DEMs. (A) Permutation test plot for OPLS-DA model: KO + MIA vs. KO + PBS. (B) Permutation test plot for OPLS-DA model: WT + MIA vs. WT + PBS. (C) Violin plots of primary metabolites for KO + MIA vs. KO + PBS. Sample size: n = 6 per group. Data show median (solid line) and IQR (density shape). Statistical significance was assessed by an unpaired Welch's t-test.


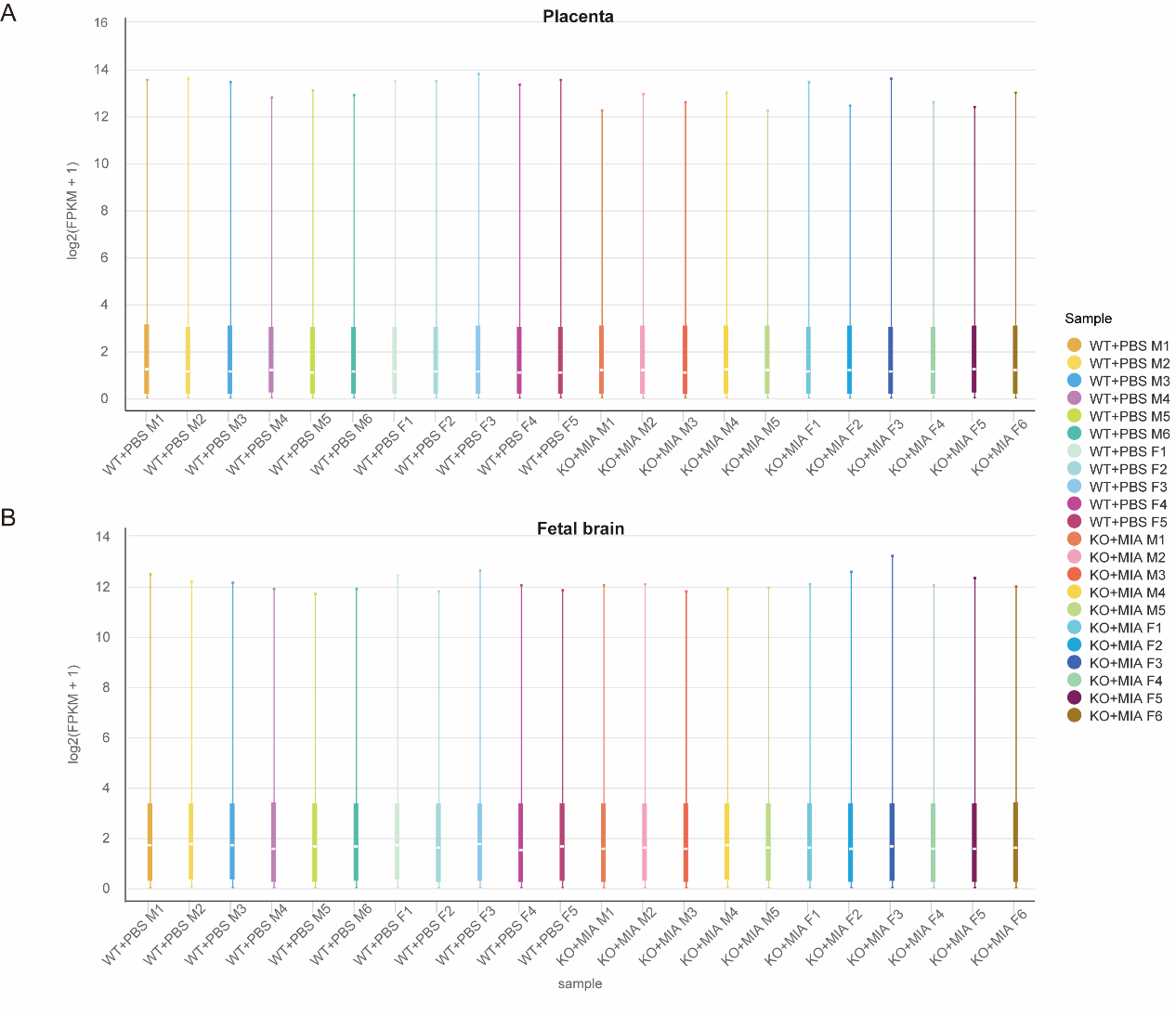


Fig. S3. Distribution of gene expression across all samples in the transcriptome. (A) Box plot of gene expression distribution across all samples in the placental transcriptome (n = 5–6). (B) Box plot of gene expression distribution across all samples in the fetal brain transcriptome (n = 5–6).


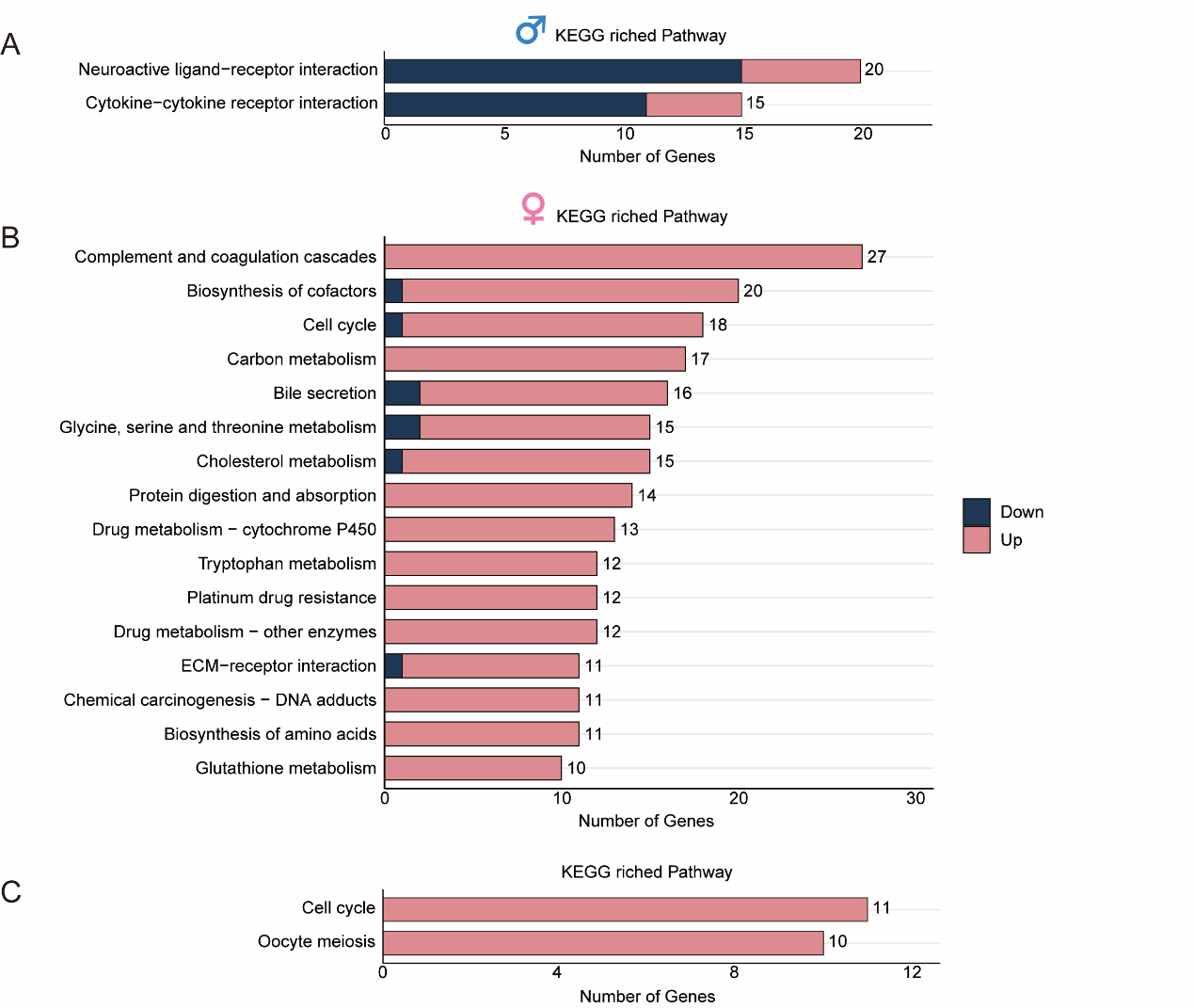


Fig. S4. Both shared and sex-specific alterations exist in male and female placentas. (A) Enriched KEGG pathways for male-specific DEGs (with > 10 associated genes). (B) Enriched KEGG pathways for female-specific DEGs (with > 10 associated genes). (C) Enriched KEGG pathways for shared DEGs (with >10 associated genes).


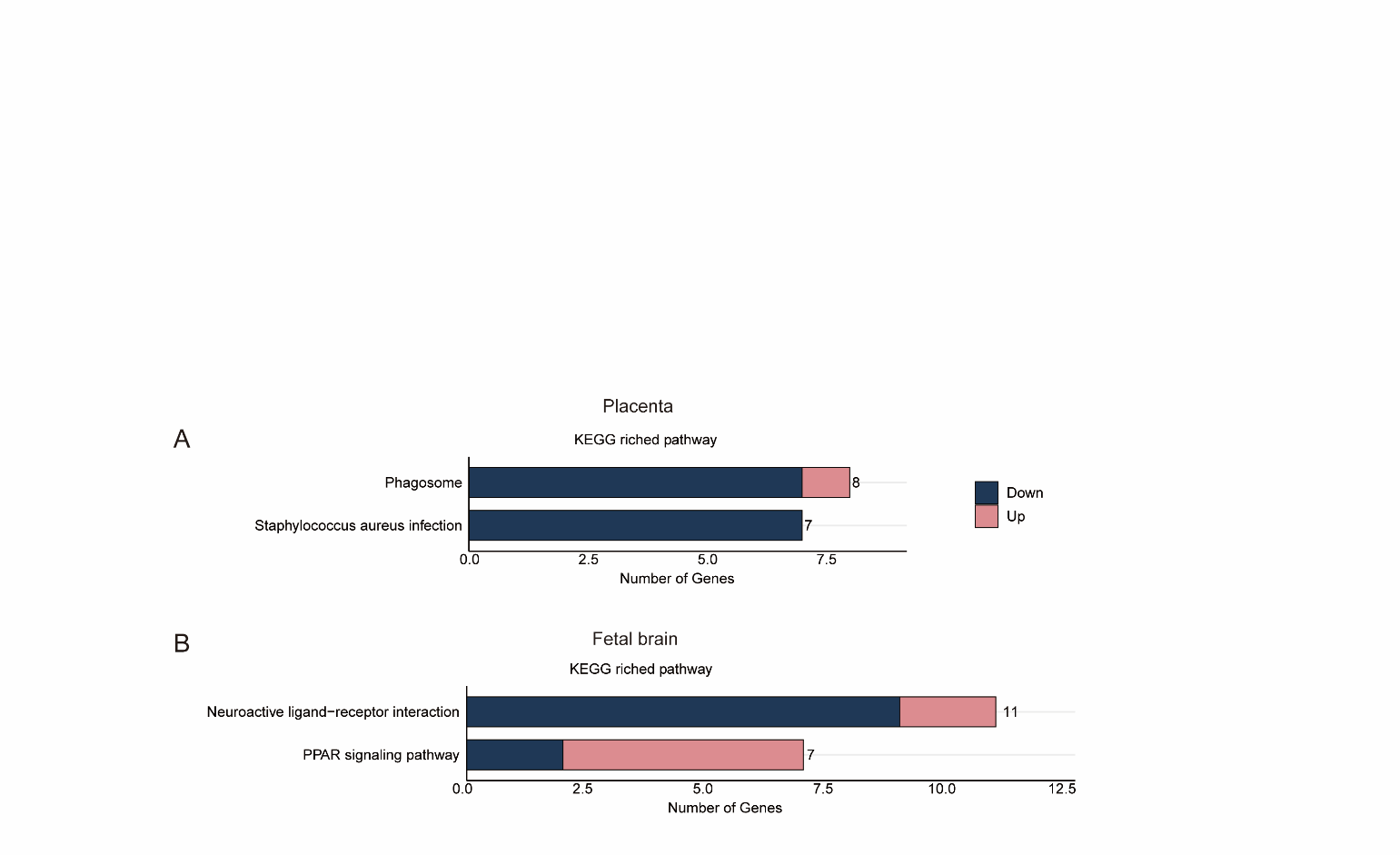


Fig. S5. Transcriptomic sex differences in double-hit placentas and fetal brains. (A) KEGG pathways enriched in DEGs from KO + MIA male vs. female placentas (with > 5 associated genes). (B) KEGG pathways enriched in DEGs from KO + MIA male vs. female fetal brains (with > 5 associated genes).
